# Polylactic Acid Nanofiber Membranes Grafted with Carbon Nanotubes with Enhanced Mechanical and Electrical Properties

**DOI:** 10.1021/acsapm.3c00776

**Published:** 2023-07-12

**Authors:** Fernando Gisbert Roca, Cristina Martínez-Ramos, Sergiy Ivashchenko, Abel García-Bernabé, Vicente Compañ, Manuel Monleón Pradas

**Affiliations:** † Center for Biomaterials and Tissue Engineering. Universitat Politècnica de València. Camino de Vera s/n, Valencia 46022, Spain; ‡ Departamento de Termodinámica Aplicada. Universitat Politècnica de València. Camino de Vera s/n, Valencia 46022, Spain; § CIBER-BBN. Biomedical Research Networking Center in Bioengineering Biomaterials and Nanomedicine. Madrid 28029, Spain; ∥ Unitat Predepartamental de Medicina, Universitat Jaume I, 12071 Castellón de la Plana, Spain

**Keywords:** polylactic acid, carbon nanotubes, electrospinning, nanofiber membranes, grafting

## Abstract

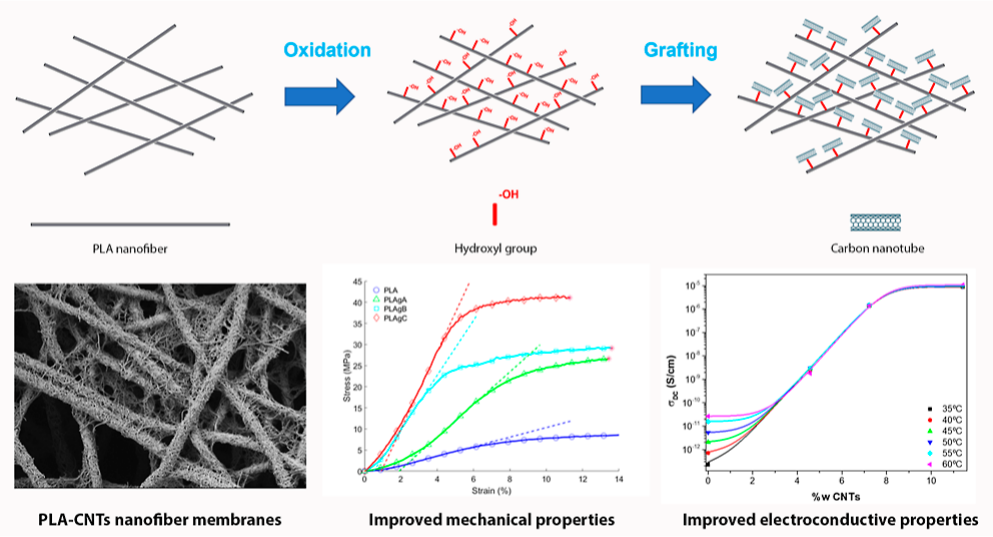

Electroconductive materials based on poly(lactic acid) (PLA) electrospinning membranes grafted with carbon nanotubes (CNTs) functionalized with the carboxylic group R–COOH have been obtained. PLA electrospun membranes were modified with sulfuric acid (H_2_SO_4_) to oxidize its surface to subsequently graft the CNTs, the treatment time and drying of the membranes before grafting with CNTs being critical, influencing the final properties of the materials. SEM images showed that CNTs presented a uniform distribution on the surface of the PLA nanofibers, while FTIR spectra of PLA-CNTs materials revealed characteristic hydroxyl groups, as evidenced by absorption peaks of CNTs. Thanks to the grafting with CNTs, the resulting PLA-CNTs membranes present an improvement of the mechanical and conductive properties when compared with PLA membranes. On the one hand, grafting with CNTs causes the nanofibers to have greater rigidity, so they are more manipulable and can more easily preserve their conformation when stress is exerted. On the other hand, grafting with CNTs allows elimination of the insulating barrier of the PLA, reducing the resistivity and providing high electrical conductivity to the PLA-CNTs membranes. The incorporation of CNTs into PLA electrospun membranes is expected to offer greater functionalities to electrospun composite nanofibers for medical and industrial applications.

## 1 Introduction

Polymer nanocomposites are considered a very promising and cost-effective technology with applications in fields like mechanical engineering, nanoscale electronics, chemical sensing, tissue engineering, and biosensing.^1,2^ In particular, conductive polymer composites (CPCs) offer electrical conductive properties with potential applications in energy storage, antistatic materials, electromagnetic interference shielding, sensors, and electrical stimulation of tissues.^1–6^ Many studies have investigated the fabrication and optimization of the surface properties of materials using conductive polymers, carbon nanotubes (CNTs), graphene-based materials, and metal or metal oxide nanoparticles. These materials are usually formed by a polymer matrix combined with one or more types of nanofillers (e.g., nanoparticles, CNTs, nanoplatelets, etc.).^7–12^

Since their discovery in 1991,^13^ CNTs have been extensively studied because of their unique advantages in terms of large specific surface area, good biocompatibility, high electrical conductivity, excellent thermomechanical properties, and good chemical stability due to their unique structure and high aspect ratio.^14–17^ Thus, CNTs have been widely applied to a large number of fields, such as advanced composites;^18–23^ energy storage and management and microelectronics;^24–28^ chemical processing;^29–31^ tissue engineering, medicine, and biosensors;^32–37^ textiles;^38,39^ aerospace;^40,41^ and environmental protection.^42^ CNTs can be prepared in different ways, such as arc discharge, chemical vapor deposition, the sol–gel method, and laser ablation. Depending on the number of graphite layers on the wall of CNTs, they can be classified into single walled CNTs (SWCNTs) and multiwalled CNTs (MWCNTs).^43^ SWCNTs have a single hexagonal sheet of carbon atoms rolled up to a tube with a diameter of 0.4–3 nm, while MWCNTs are formed by several hexagonal sheets of carbon atoms with a diameter of 4–65 nm.^44–51^

Recently, biodegradable polymers have attracted a lot of research interest because of the environmental pollution caused by petrochemical polymers. Thus, it is necessary to produce environmentally friendly CPCs based on biodegradable polymers such as polylactic acid (PLA)^52–56^ and polycaprolactone.^57–60^ This work employs PLA, which is an aliphatic polyester produced from renewable biomasses like corn and sugar beet that is a biodegradable, sustainable, and ecofriendly substituent for petroleum-based polymers. In addition, PLA has balanced properties of mechanical strength, thermal plasticity, and transparency.^61–63^ However, its crystallization rate and its thermal and mechanical properties need to be improved for long-term high-performance applications, which can be achieved by its combination with materials like CNTs, obtaining PLA-CNTs composites with enhancements in thermal, mechanical, and electrical properties.^64–67^

Another important aspect to consider is the use of nanofiber substrates based on CNTs with high porosity, large specific surface area, and interconnection of filaments, which can satisfy many biomedical and industrial requirements.^68,69^ On the one hand, the electrospinning technique is a straightforward, simple, and low-cost way to obtain nanofiber substrates which can be applied to energy storage and conversion, filtration, separation, sensors, and biomedicine.^68,70,71^ On the other hand, the surface of CNTs can be covalently functionalized with some reacting groups like –COOH, –COCl, etc., in order to react with a preformed polymer having compatible terminal functional groups like –OH and –NH_2_.^72–77^ This makes it possible to obtain electrospinning nanofiber membranes that are combined with CNTs without losing their nanoscale topography.

In this work, we obtain an environmentally friendly CPC by combining PLA and CNTs in the form of nanofiber membranes. To do this, we employ PLA electrospinning nanofiber membranes, which are treated with H_2_SO_4_ to obtain –OH groups at their surface in order to facilitate the union of CNTs functionalized with the –COOH carboxyl group. Some studies have combined PLA and CNTs, where PLA was successfully covalently grafted onto the convex surfaces and tips of MWCNTs via in situ polycondensation of l-lactic acid monomers. Other studies have incorporated CNTs into the PLA matrix obtaining bulk materials with CNTs dispersed inside.^64–67^ In this study, we obtain a PLA-CNTs CPC with a morphology based on nanofibers where PLA is used as a host polymer and CNTs are grafted on top of them to increase its mechanical and electroconductive properties. The combination of PLA and CNTs in the form of nanofibers is an innovation since normally the combination of PLA and CNTs is carried out in convex surfaces, tips, and bulk materials. In addition, we present a straightforward and effective procedure for grafting PLA fibrillar substrates with CNTs that has not been used before for this purpose. Thus, the aim of this work is to present the application of a simple method to obtain electrically conductive nanofiber membranes based on the combination of PLA fibrillar substrates and CNTs in order to employ them in applications in the fields of energy management, electronics, or tissue engineering, among others.

## 2 Experimental Section

### 2.1 Preparation of PLA Nanofiber Membranes

Using the electrospinning technique, randomly oriented PLA nanofiber membranes were obtained. PLA (INGEO 40420 RESINEX) (10 wt %) was dissolved in dichloromethane/dimethylformamide (70/30 v/v), and then the solution was stirred for 12 h at room temperature. Next, it was introduced into a 12 mL syringe (internal diameter of 15.77 mm) with a precision stainless steel needle (0.15 mm of internal diameter, 30G). The membranes were obtained by applying a voltage of 20 kV between the needle tip and the collector with a flow rate of 4 mL/h. The nanofibers were collected during 1 h on a flat plate wrapped with an aluminum foil that was placed 20 cm away from the needle tip. Finally, the membranes were air-dried for 2 days, and then they were introduced in a desiccator with fixed vacuum at room temperature for 2 days more.

### 2.2 CNTs Grafting

**Figure 1:**
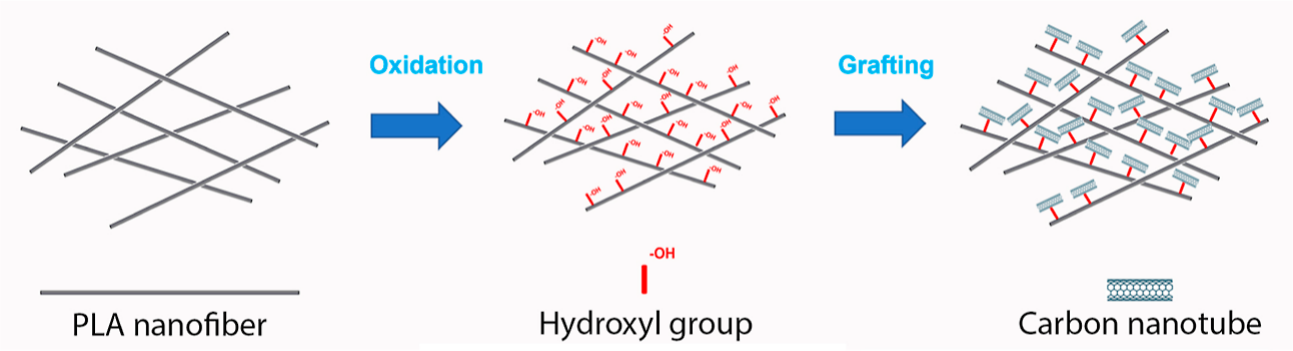
Scheme representing the steps of PLA nanofiber functionalization and grafting with CNTs.

For the first grafting protocol (PLAgA), PLA electrospinning membranes were immersed first in a 30% H_2_SO_4_ (Scharlab) solution in deionized water for 10 s (PLA10s). Then, the treated membranes were immediately immersed into a suspension of CNTs functionalized with the carboxylic group R–COOH with a diameter between 30 and 50 nm (Cheap Tubes) in sonicated deionized water (concentration of 2 mg/mL) for 3 h in a vacutainer with vacuum. Next, the membranes were washed with deionized water for 50 min under constant stirring at 350 rpm with deionized water changed every 10 min. Figure 1 represents the steps of PLA nanofiber functionalization and grafting with CNTs.

For the second grafting protocol (PLAgB), the PLA membranes were subjected to the same oxidative treatment (PLA10s) but followed by two washes in deionized water with continuous stirring for 10 min and drying in a desiccator with continuous vacuum overnight at 30 °C. Then, the membranes were immersed in a suspension of the previously described CNTs in sonicated deionized water (concentration of 2 mg/mL) for 3 h in a vacutainer with vacuum. Next, the membranes were washed with deionized water for 50 min in constant stirring at 350 rpm with deionized water change every 10 min.

For the third grafting protocol (PLAgC), the PLA membranes were immersed in a 30% H_2_SO_4_ (Scharlab) solution in deionized water for 20 s (PLA20s). Then, the treated membranes were washed twice in deionized water with continuous stirring for 10 min and dried in a desiccator with continuous vacuum overnight at 30 °C. Then, the membranes were immersed in a suspension of the previously described CNTs in sonicated deionized water (concentration of 2 mg/mL) for 3 h in a vacutainer with vacuum. Next, the membranes were washed with deionized water for 50 min under constant stirring at 350 rpm with deionized water changed every 10 min.

### 2.3 Morphological Characterization by Field Emission Scanning Electron Microscopy

In order to study the surface morphology of PLA and PLA grafted with CNTs membranes, a field emission scanning electron microscope (FESEM; ULTRA 55, ZEISS Oxford Instruments) was employed. Before imaging, samples were desiccated under vacuum for 24 h to avoid interferences caused by evaporated water. Next, samples were placed on carbon tape with a carbon bridge between each sample and the carbon tape, and the samples were grafted with a thin layer of platinum. The images were taken using a voltage of 2 kV.

### 2.4 Physicochemical Characterization

#### 2.4.1 Fourier Transform Infrared Spectroscopy Analysis

A Cary 630 Fourier transform infrared spectroscopy (FTIR) (Agilent Technologies) instrument in the attenuated total reflection mode was used to obtain FTIR spectra of the different membranes. The spectra were obtained from averages of 24 scans at 4 cm^–1^ resolution (400–4000 cm^–1^). A background measurement was carried out under the same conditions as for the baseline correction. The results represent the percentage of transmittance versus the wavenumber. To reveal significant differences between samples, different ratios of their transmittance for some particular wavenumbers were calculated. Different samples of each group were studied (*n* = 3), and the most representative spectrum of each group was represented.

#### 2.4.2 Thermogravimetric Analysis and Mass Fraction of CNTs

The thermal degradation and the composition of the different membranes were studied by thermogravimetric analysis (TGA/SDTA 851 Mettler-Toledo operated using the STARexx software). The mass loss of the samples (initial mass of approximately 2 mg) was monitored while heating up to 800 °C (10 °C/min) under a positive nitrogen flow of 20 mL/min, obtaining thermograms where the mass loss of the samples is represented as a function of temperature. Different samples of each group were studied (*n* = 3), and the most representative thermogram of each group was represented. The mass fraction of CNTs (ω_CNTs_) for the different groups was calculated from TGA residues by applying eq 1, where *m*_PLA_ and *m*_CNTs_ are the masses of PLA and CNTs at a given temperature, respectively, and ω_PLA_ is the mass fraction of PLA.
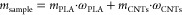
1

Due to the biphasic character of the grafted materials, ω_PLA_ = 1 – ω_CNTs_, leaving at the end only one unknown value, ω_CNTs_, which can be estimated by means of the Solver tool minimizing the sum of quadratic errors between the actual mass loss taken from the thermograms and the expected mass loss for each possible value of ω_CNTs_. Thermal degradation profiles of both neat PLA and neat CNTs served, correspondingly, as patterns for 0 and 100 wt % CNTs contents in this model.

#### 2.4.3 Differential Scanning Calorimetry

A differential scanning calorimetry (DSC) analysis of the membranes was performed using a DSC 8000 (PerkinElmer) equipped with Intracooler. Samples of approximately 2 mg were exposed to a heating cycle in the range of [40, 190] °C with a heating rate of 20 °C/min. An empty cup was used as a reference. Three different samples (*n* = 3) of each material were studied, plotting the most representative curve for each one.

The glass transition temperature (*T*_g_) was determined for each sample as the temperature at which the glass and rubber areas became most equal. These areas were delimited by three tangent lines (glass, rubber, and transition) and a vertical line corresponding to the temperature. The width of the glass transition (Δ*T*_g_) was calculated as the temperature difference between the onset and end point of the glass transition, where the onset and end point were the temperatures of the intersection of the corresponding glass/rubber tangent lines with the transition tangent line. The specific heat capacity jump at the glass transition (Δ*c*_p_) was estimated as the difference between the *c*_p_ values of glass and rubber lines at *T*_g_.

The general temperature ranges of crystallization/melting processes (Δ*T*_C_ and Δ*T*_M_) were measured as the difference between their onset and end point, the onset and end points in that case being the initial and final temperatures of the corresponding d*q*/d*T* curve deflection from the baseline applied to each thermogram. The peak temperatures of crystallization and melting (*T*_C_ and *T*_M_) were determined as the temperatures of the corresponding peaks on the thermograms. The “new crystallinity”, mass fraction of the polymer newly crystallized on heating (*X*_C_) and the “total crystallinity”, mass fraction melted during the melting process (*X*_M_), were calculated on the basis of corresponding enthalpies following eqs 2 and 3, respectively, where Δ*H*_C_ is the enthalpy of crystallization on heating, Δ*H*_M_ the enthalpy of melting (both calculated from corresponding areas from DSC thermograms), Δ*H*_f_° the heat of fusion of 100% crystalline PLA (93 J/g), and *X*_PLA_ the mass fraction of PLA in the materials. The mass fraction of PLA in the samples was calculated from TGA data (1 for neat PLA and treated samples, 0.9543 for PLAgA, 0.9278 for PLAgB, and 0.8859 for PLAgC samples). The “initial crystallinity”, mass fraction of the previously existent crystalline phase (*X*_0_) before doing DSC, was calculated as the difference, *X*_M_ – *X*_C_.
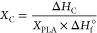
2
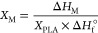
3

### 2.5 Mechanical Properties of the Membranes

The mechanical properties were characterized by tensile tests with a Universal Testing Machine (UTM) Microtest (Microtest, Madrid, Spain) displacement control to obtain force data (N), position (mm), and time (s). It was equipped with a 15 N load cell with Microtest SCM3000 95 software (Microtest, Madrid, Spain) (force resolution 0.001 N, position 0.001 mm, and time 0.1 s). Data were recorded every 0.5 s and subsequently exported to Excel (Microsoft Excel for Mac, Version 16.23, Redmond, WA). Rectangular membranes were clamped in the UTM with sandpaper between the clamp and sample to avoid slipping. The nanofibers were always parallel to the stretching direction. All pieces were stretched at a displacement rate of 1 mm/min up to breaking, and the stress (σ) was calculated for each measured force value (*F*), as the quotient between the force and the cross section of the sample (*A*) (*A* = *e*·*b*; ), where *e* and *b* are, respectively, the thickness and width of the sample. For each recorded point, the deformation of the sample (Δ*l*), as the difference between the position at that point (*l*) and the initial position (*l*_0_) (Δ*l* = |*l* – *l*_0_|), and the strain (ε) () were also calculated. A graph of stress against strain was built for each test, and Young’s modulus of the sample was calculated as the slope of the curve in the linear zone. To do it, a linear fitting of those points lying along a straight line was carried out with an Excel spreadsheet. The breaking point was taken at that point where the force suddenly decreased.

### 2.6 Resistivity Properties

The electrical properties of the PLA membranes before and after grafting with CNTs were studied by measuring superficially the circulating electric current (dc) when applying a known voltage, calculating the apparent surface electrical resistance of the materials (*R*) by Ohm’s law. Measuring the distance between contacts (*l*) and the cross section of the membranes (*S*), the in-plane apparent resistivity (ρ) of the materials was obtained by eq 4. Rectangular membranes with dimensions of 8 × 5 mm were employed. The distance between the contact points was 8 mm. Three different samples (*n* = 3) of each material were studied.
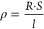
4

### 2.7 Electrochemical Impedance Spectroscopy

A Novocontrol Broadband Dielectric Spectrometer (Hundsangen, Germany) integrated with an SR 830 lock-in amplifier with an α dielectric interface was employed to measure the complex conductivity and the permittivity of the PLA membranes grafted with CNTs by impedance spectroscopy at different temperatures between 273 K (0 °C) and 333 K (60 °C) with a frequency window of 10^–1^ < *f* < 10^7^ Hz. The samples were placed between two gold electrodes, and the experiments were carried out with 100 mV amplitude. When the conductivity measurements were performed, the temperature was maintained isothermally or changed stepwise within the temperature window controlled by a nitrogen jet (QUATRO from Novocontrol) with a temperature error of 0.1 K during every single scan in frequency.

### 2.8 Statistical Analysis

The GraphPad Prism software was employed for the statistical analysis of the results. The one-way ANOVA test together with a multiple sample mean comparison (Tukey’s multiple comparisons test with a significance degree of 95%) was employed to study the presence of differences between groups. Statistically significant differences are indicated by *, **, ***, or **** (*p*-value below 0.05, 0.01, 0.001, or 0.0001, respectively). Results are expressed as the mean ± standard deviation (SD) or as the mean ± standard error of the mean (SEM).

## 3 Results

### 3.1 H_2_SO_4_ Treatment and CNTs Grafts Characterization and Morphological Properties

**Figure 2:**
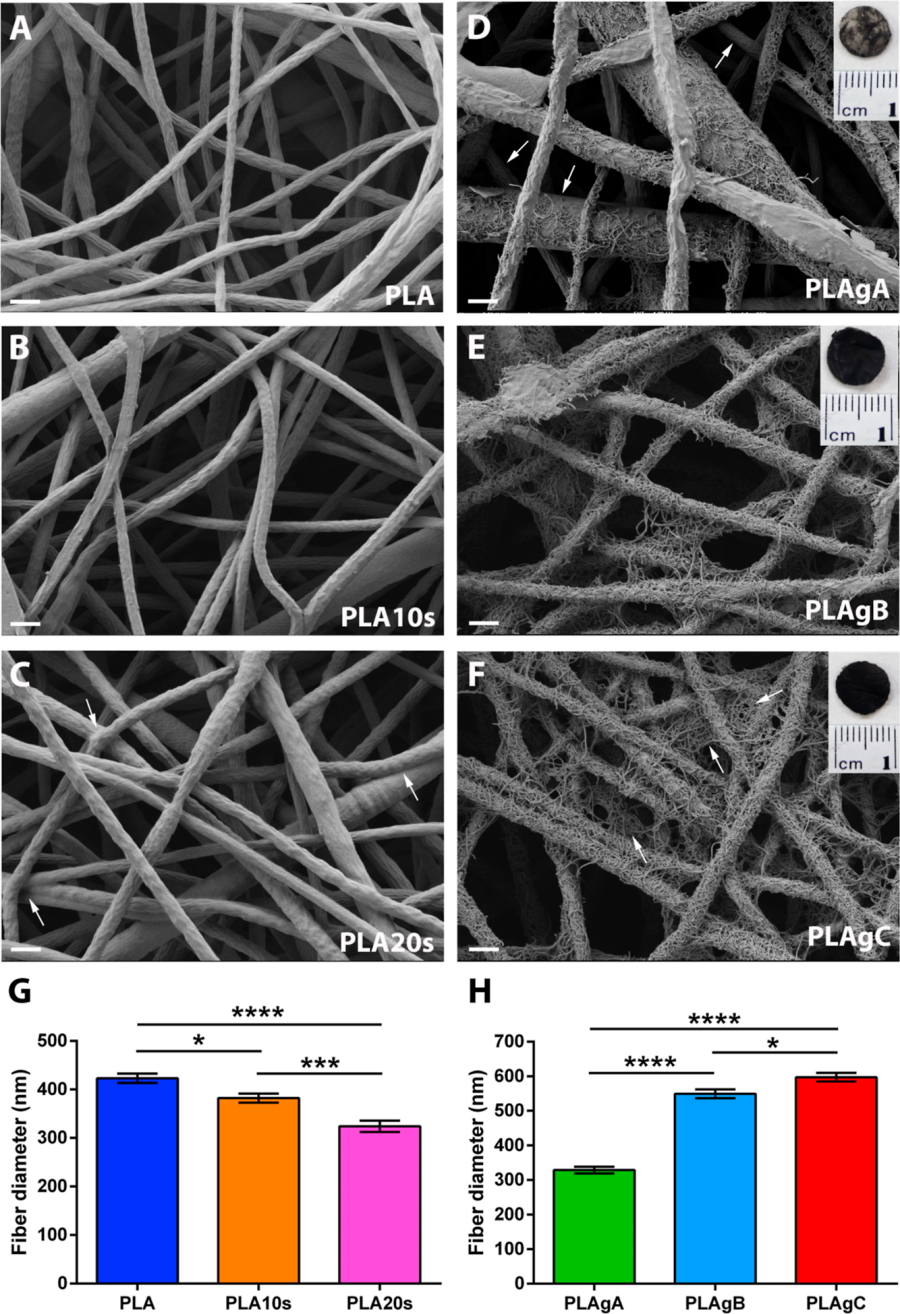
Images of the membranes and characterization of their fiber diameter before and after CNTs grafting. (A) FESEM image of a nontreated PLA electrospinning membrane. (B) FESEM image of a PLA electrospinning membrane treated with 30% H_2_SO_4_ for 10 s. (C) FESEM image of a PLA electrospinning membrane treated with 30% H_2_SO_4_ for 20 s. Arrows indicate the fusion of some nanofibers due to H_2_SO_4_ treatment. (D) FESEM and macroscopic (insert) image of a PLA electrospinning membrane grafted with CNTs following the grafting protocol A. The arrows indicate areas without CNTs. (E) FESEM and macroscopic (insert) image of a PLA electrospinning membrane grafted with CNTs following the grafting protocol B. (F) FESEM and macroscopic (insert) image of a PLA electrospinning membrane grafted with CNTs following the grafting protocol PLAgC. The arrows indicate areas where the CNTs fill the gaps between nanofibers. (G) Fiber diameter of nontreated PLA membranes (PLA) and of 30% H_2_SO_4_-treated PLA membranes for 10 and 20 s (PLA10s and PLA20s, respectively). (H) Fiber diameter of PLA membranes grafted with CNTs following the grafting protocols A, B, and C (PLAgA, PLAgB, and PLAgC, respectively).

First, the microscopic effect on the electrospinning membranes of immersion in 30% H_2_SO_4_ for 10 s (PLA10s) and for 20 s (PLA20s) was studied by means of FESEM images (Figure 2A–C). Regarding the samples with the 10 s treatment (Figure 2B), their nanofibers did not show significant differences in the surface morphology with respect to the control samples (Figure 2A). However, in the ultrastructure of the samples subjected to 20 s (Figure 2C), it was observed that the nanofibers acquired a less smooth surface, characterized by micrometric grooves and valleys, in addition to a fusion tendency between fibers.

The samples grafted with CNTs were also observed by means of FESEM images (Figure 2). For the PLAgA samples (Figure 2D), it was observed that different areas did not present CNTS, indicating a deficient and nonhomogeneous grafting of the membranes. However, for the PLAgB and PLAgC samples, the surface was full of nanotubes, apparently with a greater amount in PLAgC, with the formation of lattices as a connective network between nanofibers (Figure 2E,F). This is consistent with what was observed macroscopically since PLAgA presented a grayish appearance and a surface with accentuated roughness, while PLAgB and PLAgC showed a surface entirely black with a smooth texture (details of Figure 2, macroscopic photos).

The quantification of the diameter of the fibers (Figure 2G,H) revealed that the fibers treated with H_2_SO_4_ presented a decrease in diameter when compared with the untreated PLA. This decrease was greater on average for the 20 s treatment, which can be due to its longer exposure to H_2_SO_4_, confirming that the reagent attacks by degrading the PLA surface. When the treated nanofibers are grafted with CNTs, there was a notable increase in the diameter of the fibers, with an average increase of approximately 165 nm for PLAgB and 270 nm for PLAgC. However, the grafting generates in both groups approximately a value of 550–600 nm in diameter, so there are no differences, although they presented a diameter increase of 220 nm with respect to the PLAgA group.

### 3.2 Physicochemical Characterization

**Figure 3:**
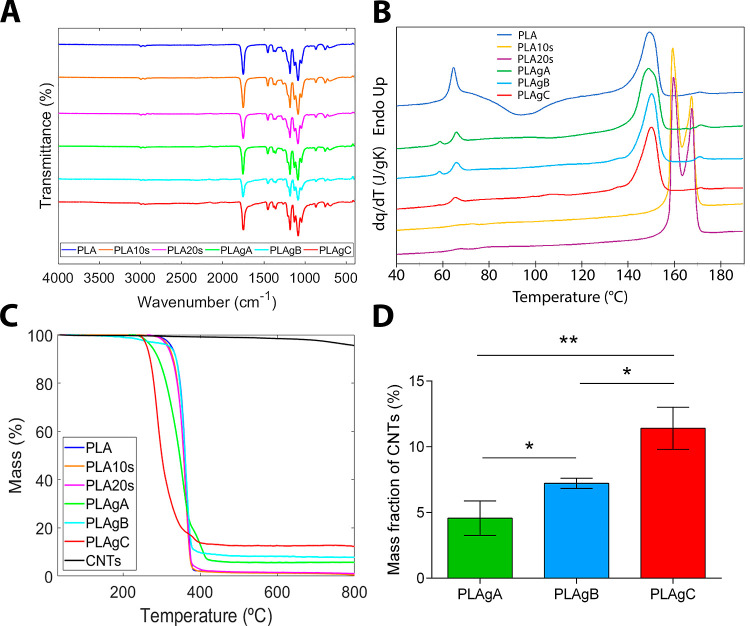
(A) FTIR diagrams of all samples, representing transmittance as a function of wavenumber. (B) DSC thermograms of the samples represent normalized heat flow on heating with a temperature. (C) TGA thermograms of neat PLA, CNTs, treated samples, and grafted with CNTs, representing residual weight with a temperature. (D) CNTs contents in grafted samples were calculated from profiles of their thermal degradation.

The FTIR data obtained from PLA, PLAgA, PLAgB, and PLAgC membranes apparently present the same transmittance signal, with a very limited variability between the replicates of each sample (Figure 3A). Nevertheless, the ratio values for most relevant transmittance peaks show significant differences between the samples (Figure S1A,B; Table S1); the most important differences were observed at 1759 cm^–1^ (corresponding to the C=O stretch), at 1181 cm^–1^ (corresponding to the asymmetric C=O stretch in the ester group), and at 1084 cm^–1^ (corresponding to the asymmetric C=O stretch in the ether group). Generally, while ratios PLA10s/PLA were lying between 0.96 and 0.99, the values for PLA20s/PLA were lying between 1.02 and 1.17, revealing significantly higher transmittance and lower absorbance in the PLAgC sample than in the PLA. Hence, it can be concluded that longer time (20 vs 10 s) of exposure to H_2_SO_4_ caused a pronounced modification in the chemical structure of PLA, e.g., decrease in number of C–O bonds. This chemical alteration allows the carbon atom to acquire a negative charge due to the effect of the resulting unpaired electron, thus allowing the carboxyl group of the CNT to join via condensation to the so-functionalized PLA, forming a stable covalent bonded macrostructure. A similar but even more pronounced effect was observed for PLAgA and PLAgB samples compared to the simply treated ones: values of 1.02–1.09 for PLAgA/PLA10s ratio and values of 1.07–1.53 for PLAgB/PLA10s ratio may reflect an important decrease in the number of related groups due to covalent bonding of CNTs or attenuating effect of proper CNTs layer covering PLA. However, such effect was not observed on PLAgC samples, with values of 0.89–1.02 for PLAgC/PLA20s ratio, possibly due to a contribution of chemical groups provided with CNTs.

The absence of sulfur in H_2_SO_4_-treated membranes was assessed with the FTIR spectra of PLA10s and PLA20s samples. Since none of the peaks corresponding to the functional groups of elemental sulfur described in ref (78) were observed in the FTIR spectra of PLA10s and PLA20s samples, we could conclude that no traces of sulfur were left on the PLA membranes. This can be explained by the fact that the sulfuric acid used for the oxidation of PLA nanofibers is highly diluted in water and that after the treatment with the sulfuric acid solution the samples are washed twice in deionized water and dried in a desiccator with continuous vacuum.

The DSC thermograms of all of the compositions are shown in Figure 3B. PLA samples undergo a well-developed glass transition, starting (onset) at 53 °C, reaching glass transition temperature (*T*_g_) around 58 °C, and being completed up by 61 °C, showing a width (Δ*T*_g_) of about 8 °C and a heat capacity jump (Δ*c*_p_) of about 0.218 J g^–1^ K^–1^. In acid-treated samples (PLA10s and PLA20s), glass transition is shifted to higher temperatures, starting between 56 and 60 °C, reaching *T*_g_ around 60 °C for PLA10s and 69 °C for PLA20s, and being completed by 80 °C, showing much broader Δ*T*_g_ (between 19 and 24 °C) and significantly reduced Δ*c*_p_ (0.013 J g^–1^ K^–1^ for PLA10s and 0.142 J g^–1^ K^–1^ for PLA20s). In CNTs-grafted samples, glass transition starts between 52 and 54 °C, reaches *T*_g_ between 58 and 61 °C (following in both cases the tendency PLAgA < PLAgB < PLAgC) and is completed by 63 °C, with a slight reduction of Δ*T*_g_ (11 °C for PLAgA, 9 °C for PLAgB, and 8 °C for PLAgC samples), while Δ*c*_p_ shows a significant reduction (0.147 J g^–1^ K^–1^ for PLAgA, 0.132 J g^–1^ K^–1^ for PLAgB, and 0.109 J g^–1^ K^–1^ for PLAgC). The process of crystallization during the heating in neat PLA takes place between 77 and 119 °C, resulting in up to 18% of polymer mass newly crystallized (*X*_C_) from amorphous state, but this process is drastically suppressed in both treated and grafted samples, giving an X_C_ of only up to 4% (nearly 0% for PLA20s and PLAgB samples). Melting in PLA samples is a single-step process that takes place between 120 and 160 °C (with the peak temperature, *T*_M_, at 149 °C), resulting in 29% of polymer mass totally molten (*X*_M_) from the crystalline state, which means initial crystallinity (*X*_0_) of PLA was around 11%. In treated samples (PLA10s and PLA20s), melting takes place between 150 and 180 °C and is a double-step process, presenting its first peak, *T*_M1_, around 160 °C and the second, *T*_M2_, around 168 °C, with *X*_M_ reaching 51–52% of PLA mass, which almost entirely corresponds to *X*_0_. In grafted samples (PLAgA, PLAgB, and PLAgC), crystalline phase melting is a single-step process that takes place between 128 and 160 °C, with *T*_M_ values of about 149–150 °C and *X*_M_ values of about 27–29%, mostly corresponding to *X*_0_. The observed differences in *X*_0_ are represented in Figure S2.

Such differences in crystallinity and crystallization profiles between the treated samples (PLA10s and PLA20s) and PLA could be explained by the effect of acidic treatment which introduces heterogeneities and covalently modifies the surface of PLA membranes through the formation of oxides and other species that could directly stiffen the amorphous phase and, furthermore, incites its important structural reorganization, with transition from more amorphous to a more crystalline pattern.^79^ Moreover, during the treatment, H^+^ ions predominantly penetrate the less ordered amorphous polymeric regions, cleaving sensitive bonds while leaving intact more resistant crystalline regions,^80,81^ hence causing a reduction of amorphous PLA fraction, with consequent increase of the relative crystallinity in treated samples. Besides, partial cleavage of chains can provoke recrystallization events in the amorphous phase. The considerable decrease of Δ*c*_p_ at *T*_g_ detected in treated and grafted samples (comparing with PLA) reflects stiffening and proportional decrease of their amorphous phase capable of undergoing glass transition and is intensified with additional restrictions on PLA chains’ molecular mobility by the increased crystalline phase (higher *X*_0_) after acidic treatment. In addition, the significant increase of Δ*T*_g_ detected in treated samples may reflect the corresponding increase in heterogeneity of PLA domains undergoing glass transition, caused by the proper treatment itself, the effect of which appears to be softened with further grafting. The effect on proper *T*_g_, however, is quite slight as the described changes are of more superficial than bulk nature.

The different melting profile in treated membranes, especially considering its shift to higher temperatures, should also be attributed to the mentioned structural reorganization and higher crystallinity in treated samples that enhances their thermal stability and postpones the melting process at more than 10 °C. Its double-step progress can be explained with two types of crystals present in PLA: one previously existent and one additional, formed due to the acidic treatment (according to the above-described alterations of the amorphous phase). Nevertheless, in CNTs-grafted samples, these effects appear to be softened, as the grafting process consumes the reactive species created by acidic treatment and interrupts PLA chains cleavage in the amorphous phase. As a result, the further chains rearrangement and recrystallization events are suppressed, limiting the resulting crystallinity (*X*_0_) in grafted PLA membranes and preventing considerable formation of new type of crystals, thus “restoring” a single-step appearance and earlier onset of the melting process.

The TGA in Figure 3C represents the loss of mass as a function of temperature for neat PLA, treated, and grafted samples. Thermal degradation of neat PLA is a simple one-step process, taking place between 300 and 442 °C (with a maximum rate at ≈365 °C). Treatment with sulfuric acid does not affect this process much but slightly accelerates its beginning: in 10 s-treated samples, thermal degradation begins at 295 °C, and in 20 s-treated samples, at 290 °C, probably as a consequence of partial loss of integrity on the surface of PLA microfiber due to the treatment. However, grafted materials show more complex and variable profiles of their thermal degradation, with significant differences between samples. In PLAgA membranes, it is a double-step process: the first step takes place between 244 and 260 and 375 °C, resulting in a 70–85 wt % mass loss, while the second step occurs between 377 and 446 °C, resulting in a 6–23 wt % mass loss. In PLAgB membranes, thermal degradation profiles express much lesser variability, and the first step is notably reduced: being concluded between 241 and 286 °C, it results in only 2–3 wt % mass loss, while the second step is more substantial, contributing up to 87 wt % mass loss between 319 and 443 °C. PLAgC membranes demonstrate higher variability of thermal degradation: the first step happens between 240 and 334–359 °C, resulting in a 77–82 wt % mass loss, and the second step takes place between 337 and 361 and 432–444 °C, resulting in a 4–10 wt % mass loss.

Generally, TGA curves reveal the shift of thermal degradation onset to lower temperatures in the presence of CNTs, following the next pattern: PLAgC < PLAgB < PLAgA ≪ PLA20s < PLA10s < PLA. This can be explained because (1) the treatment with more concentrated acid (in cases of PLAgC and PLA20s membranes) leads to greater diminution in the diameter of the microfiber compared to that in PLAgA, PLAgB, and PLA10s samples and (2) the higher thermal conductivity of CNTs may help in heat transfer into the polymer matrix and accelerate the early onset of degradation.

Another observed phenomenon is a significant difference in residual weight between types of samples after completing their thermal degradation (after 450 °C): the lowest values, about 1–2 wt %, were detected in neat PLA and acid-treated samples, subsequently increasing in grafted samples (PLAgA < PLAgB < PLAgC): 5.5–6.8 wt % for PLAgA, 8.6–9.2 wt % for PLAgB, and 12.2–14.1 wt % for PLAgC ones. As the thermal stability of CNTs under a nitrogen atmosphere is very high (Figure 3C), samples grafted with CNTs undergo less thermal degradation than uncoated PLA and leave more residues. The separate study of neat CNTs thermal degradation performed under the same conditions confirmed that they do not suffer significant mass loss (up to 2.27 wt % as much) until 700 °C, always leaving the final residue higher than 95 wt % at 800 °C.

TGA residues suggest that the possible CNTs contents in grafted samples may also follow the trend PLAgA < PLAgB < PLAgC. It was successfully confirmed by indirect estimation based on profiles of their thermal degradation (according to eq 1) in the range between 450 and 700 °C (Figure 3D). The data obtained show the effectiveness of the grafting process, being somewhat lesser for grafting A, higher for grafting B, and considerably higher for grafting C, which is in conformity with the results obtained in other characterization tests. Actually, PLAgA membranes were treated with H_2_SO_4_ for 10 s without further overnight drying, being directly subjected to grafting with CNTs instead, so that the resultant oxidative layer was not firmly settled on their microfibers surface. In the PLAgB and PLAgC samples, the H_2_SO_4_ treatment was immediately followed by the washing step and subsequent overnight drying that perhaps allowed the oxidative layer to adhere well to their microfiber surface, resulting in higher regularity in the distribution of CNTs at the time of grafting.

A comparative analysis of neat PLA, grafted samples, and neat CNTs thermal degradation between 450 and 700 °C allowed us (as described in Experimental Section) to estimate a row CNTs contents in grafted materials as 4.57 ± 1.31 wt % for PLAgA, 7.22 ± 0.39 wt % for PLAgB, and 11.41 ± 1.60 wt % for PLAgC (Figure 3D), hence following the growing trend PLAgA < PLAgB < PLAgC. A single factor one-way ANOVA test provided *F* > *F*_critical_ (48.44 > 5.14), therefore rejecting the null hypothesis about equality of these means and suggesting significant differences in estimated CNTs contents between PLAgA, PLAgB, and PLAgC materials. Furthermore, the two-sample *t*-test (assuming unequal variances) was performed in pairs PLAgA/PLAgB, PLAgA/PLAgC, and PLAgB/PLAgC, providing *t*_stat_ < −*t*_critical two-tail_ for all compared pairs (−4.76 ← 4.30 for PLAgA/PLAgB, −8.11 ← 2.78 for PLAgA/PLAgC, and −6.23 < −4.30 for PLAgB/PLAgC), and thus confirming the CNTs contents between three types of grafting differ significantly. The smallest difference is detected between PLAgA and PLAgB samples, the medium-between PLAgB and PLAgC, while the highest difference was noted between PLAgA and PLAgC materials. These results exhibit that the PLAgC process had the highest efficiency of CNTs grafting on PLA, and the PLAgA had the lowest one.

### 3.3 Mechanical Testing

**Figure 4:**
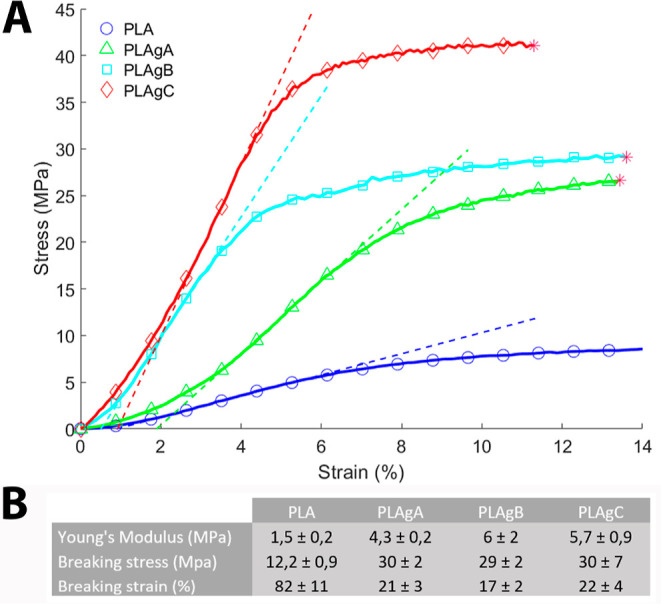
A) Tensile tests. (B) Parameters obtained from the tensile tests.

From the results (stress–strain curves, Figure 4), it can be observed that the stiffness increases with the content of CNTs (increase of Young’s modulus). The correlation coefficient of the linear fitting is near 1 in every case, validating the linearity of the behavior in that zone. The PLA sample without CNTs shows less Young’s modulus, gradually increasing on coated samples PLAgA, PLAgB, and PLAgC.

Moreover, the samples coated with CNTs show a higher breaking stress (PLAgA, PLAgB, and PLAgC) than the sample without CNTs (PLA) probably because of the reinforcing effect of CNTs. Regarding the breaking strain, the higher the CNTs content, the lower the breaking strain. This behavior can be explained by the treatment with H_2_SO_4_, which could induce the fragility of the samples. In addition, the stress over the grafting can contribute to the constriction of nanofibers by transferring accumulated loads.

### 3.4 Resistivity in Plane

**Figure 5:**
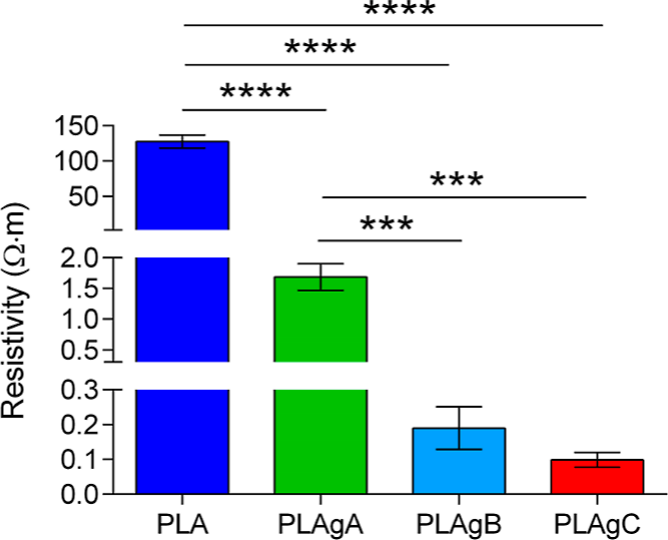
Resistivity values obtained from PLA electrospinning membranes before and after the grafting with CNTs (PLA, PLAgA, PLAgB, and PLAgC).

The resistivity values, measured using the in-plane method described in Section 2.6, are listed in Figure 5. From this figure, we can see that the uncoated PLA membranes presented a very high resistivity when compared to the other groups. For this reason, grafting with CNTs is necessary in order to decrease the resistivity value of the PLA membranes. As can be observed, PLA-gA samples presented a notable drop in resistivity, although they did not reach the even lower values of PLAgB and PLAgC samples. These differences may be due to the fact that the CNTs grafting of PLAgA samples was not homogeneous and therefore the PLAgA membranes had a lower content of CNTs, so that the conductivity along the rectangular membrane was heterogeneous. However, for the PLAgB and PLAgC samples, the CNTs grafting appearance was quite homogeneous in comparison with PLAgA, with a little more content of CNTs in PLAgC. The values of in-plane electrical conductivity follows the trend: σ_DC_ (PLA) = 80 × 10^–6^ ± 6 × 10^–6^ S/cm < σ_DC_ (PLAgA) = 62 × 10^–4^ ± 8 × 10^–4^ S/cm < σ_DC_ (PLAgB) = 8 × 10^–2^ ± 3 × 10^–2^ S/cm < σ_DC_ (PLAgC) = 12 × 10^–2^ ± 3 × 10^–2^ S/cm.

### 3.5 Dielectric Properties

**Figure 6:**
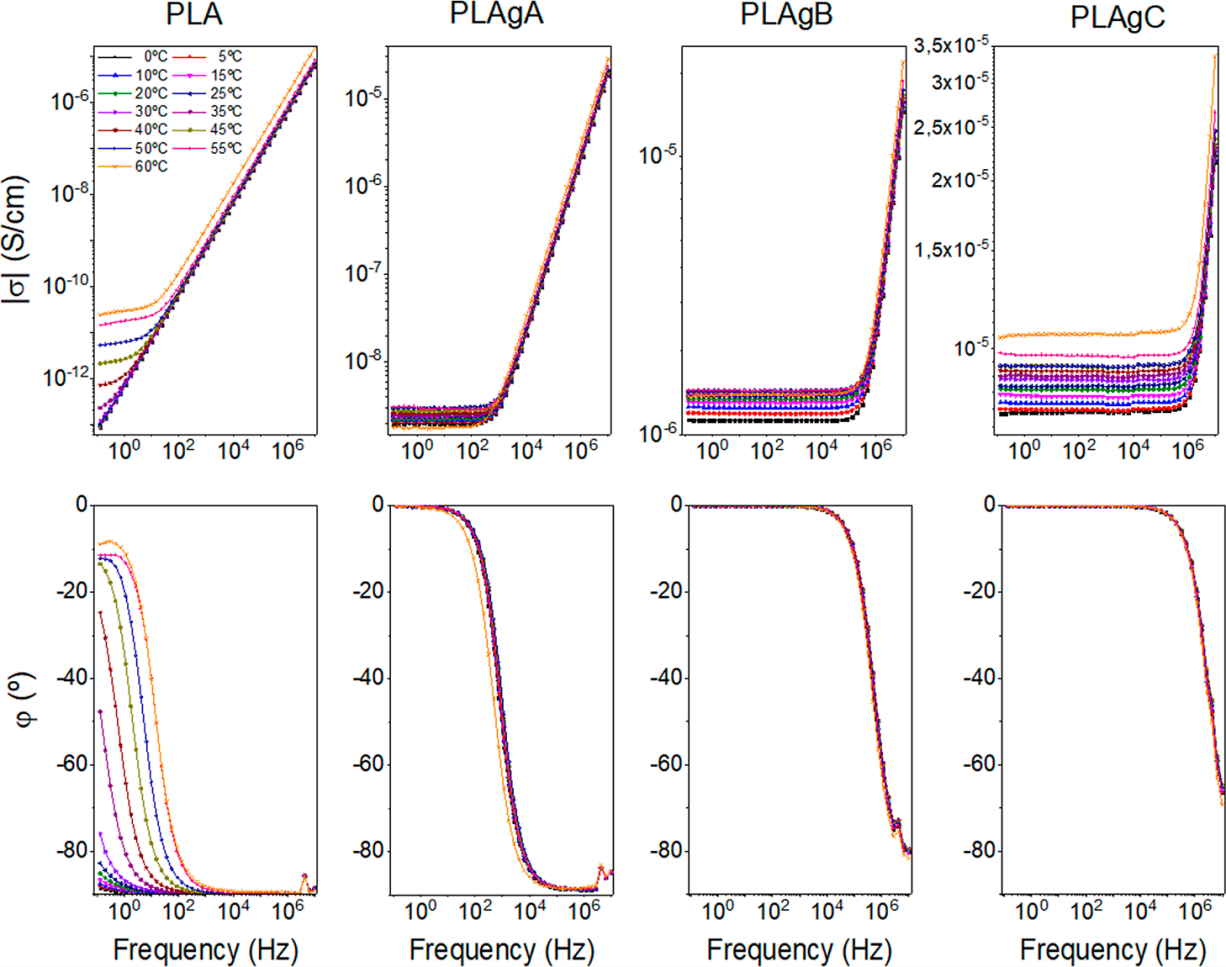
Bode diagrams for PLA, PLAgA, PLAgB, and PLAgC samples. (Up) Modulus of conductivity vs frequency at different temperatures. (Down) Phase angle vs frequency at different temperatures.

Electrochemical impedance spectroscopy measurements were carried out for PLA, PLAgA, PLAgB, and PLAgC samples at different temperatures (0–60 to 60 °C with a step of 10 °C) to obtain information on the through-plane conductivity of the samples. For this, data for the real part of the conductivity were analyzed in terms of the corresponding Bode diagrams, where conductivity variations with frequency are obtained for all groups, as shown in Figure 6 (up), and the phase angles are represented below in Figure 6 (down). From these panels, we can see that when the phase angle tends to zero the dc-conductivity is independent of the frequency for each temperature. The conductivity values (σ = σ_dc_) were obtained at the frequency where the phase angle φ(deg) is zero.

When the PLA membranes are modified adding different percentages of CNTs (PLAgA, PLAgB, and PLAgC), an increase of the polarity appears when compared with the base-polymer (PLA). This can be a possible explanation for the behavior of PLA-CNTs samples. In the case of PLAgA, the conductivity values are significantly lower than in the case of PLAgB and PLAgC. Thus, for this case, the sample has a behavior different from that of a material purely capacitive or resistive. This can be explained as a Debye relaxation due to the macroscopic polarization of the charges as a consequence of the applied electric field. This relaxation is characterized by a relaxation time, which depends on the temperature, chemical structure of the samples, and their thickness. This behavior may be due to the reorientation motion of dipoles and more likely to the motion of the localized charges, which dominate the dc-conductivity.^82–89^ Similar observations can be made for the PLA sample, where the real part of the conductivity is also constant at the low-frequency region until a cutoff frequency where it starts increasing with the frequency as if the sample was a capacitor. When the conductivity values are constant with the frequency, it means that the impedance has only a resistive contribution, and its value represents the electrical conductivity of the sample. The conductivity value for each one of the samples can be obtained from the intercept in the *OY*-axis (i.e., from the intersection of the extrapolated frequency-independent plateau line).

From Figure 6, we can observe that the dc-conductivity measured through the plane increases when the temperature increases and when the mass fraction of CNTs increases. For example, at 40 °C the conductivity follows the trend: σ_DC_ (PLA) = 7.1 × 10^–13^ S/cm < σ_DC_ (PLAgA) = 2.6 × 10^–9^ S/cm < σ_DC_ (PLAgB) = 1.4 × 10^–6^ S/cm < σ_DC_ (PLAgC) = 9.1 × 10^–6^ S/cm. These values show the same trend as the values observed in the in-plane measurements, such as those commented on above in Section 3.4.

On the other hand, a close inspection of Figure 6 allows to observe that the samples present a behavior similar to that of a capacitor when the amount of CNTs is low and intermediate (samples PLAgA and PLAgB), where the phase angle is −90° or tend to this value at high frequencies.

Regarding the conductivity, Figure S6 depicts the dependence of the dc-conductivity on temperature for the PLA samples doped with different amounts of CNTs. An Arrhenius behavior was observed for all groups. The activation energy (*E*_a_) follows the trend: *E*_a_ (PLA) = 176.3 kJ/mol > *E*_a_ (PLAgA) = 5.2 kJ/mol > *E*_a_ (PLAgB) = 3.2 kJ/mol > *E*_a_ (PLAgC) = 3.1 kJ/mol. This tendency is also shown in Figure S6-inset. These values were calculated in the temperature range between 0 and 55 °C for the PLA-CNTs groups and between 35 and 55 °C for the PLA group.

Regarding the behavior of Bode diagrams shown in Figure 6, we observe that the samples present a typical behavior of a parallel RC circuit (see Supporting Information). For the PLA group, we can conclude that the electrical response in the range of frequencies between 1 and 10^7^ Hz is purely capacitive, where the cut-frequency varies with temperature. For the PLAgA group, the range of frequencies where the sample maintains a capacitive behavior changes to the interval between 10^3^ and 10^7^ Hz. For the PLAgB group, this range is observed from 10^5^ to 10^7^ Hz. Finally, for the PLAgC group, the behavior is practically resistive in all ranges of frequencies. Notice that for the three PLA-CNTs groups, the cutoff frequency is practically independent of temperature, contrary to what happens with the PLA group, where the cutoff frequency varies with the temperature.

For the PLAgC group, the conductivity in the Bode diagram tends to a constant value in practically all of the regions of frequencies, which means that the sample presents a conductive behavior. We can observe the values of conductivity by the intersection of the conductivity modulus when frequency tends to zero (between 10^–7^ and 10^–5^ S/cm, depending on temperature), which are similar to those obtained by other studies where PLA is combined with CNTs, as shown in Table 1.

**Table 1:** Comparison of the Conductivity of Samples Grafted with CNTs (PLAgA, PLAgB, and PLAgC) and PLA/CNTs Composites from Other Studies

comparison of the electrical properties of PLA/CNT composites		
reference	CNTs content (wt %)	conductivity (S/cm)
PLAgA	4.6	10^–9^
PLAgB	7.2	10^–6^
PLAgC	11.4	10^–5^
(52)	2.0	10^–7^
(64)	3	10^–5^
(67)	1.2	10^–6^
(90)	1.2	10^–9^
(91)	1.2	10^–6^
(92)	1.0	10–^4^

There is a diminishing of these conductivity values when the mass fraction of CNTs decreases (PLAgB, PLAgA, and PLA), as seen before. At the same time, the phase angle in the low-frequency region increases from −90 for the PLA group to 0 for the PLA-CNTs groups. The critical frequency was taken as the value at which the modulus of the conductivity is constant.

We have calculated the capacity of the samples from the complex permittivity, considering that
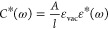
5
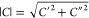
6

**Figure 7:**
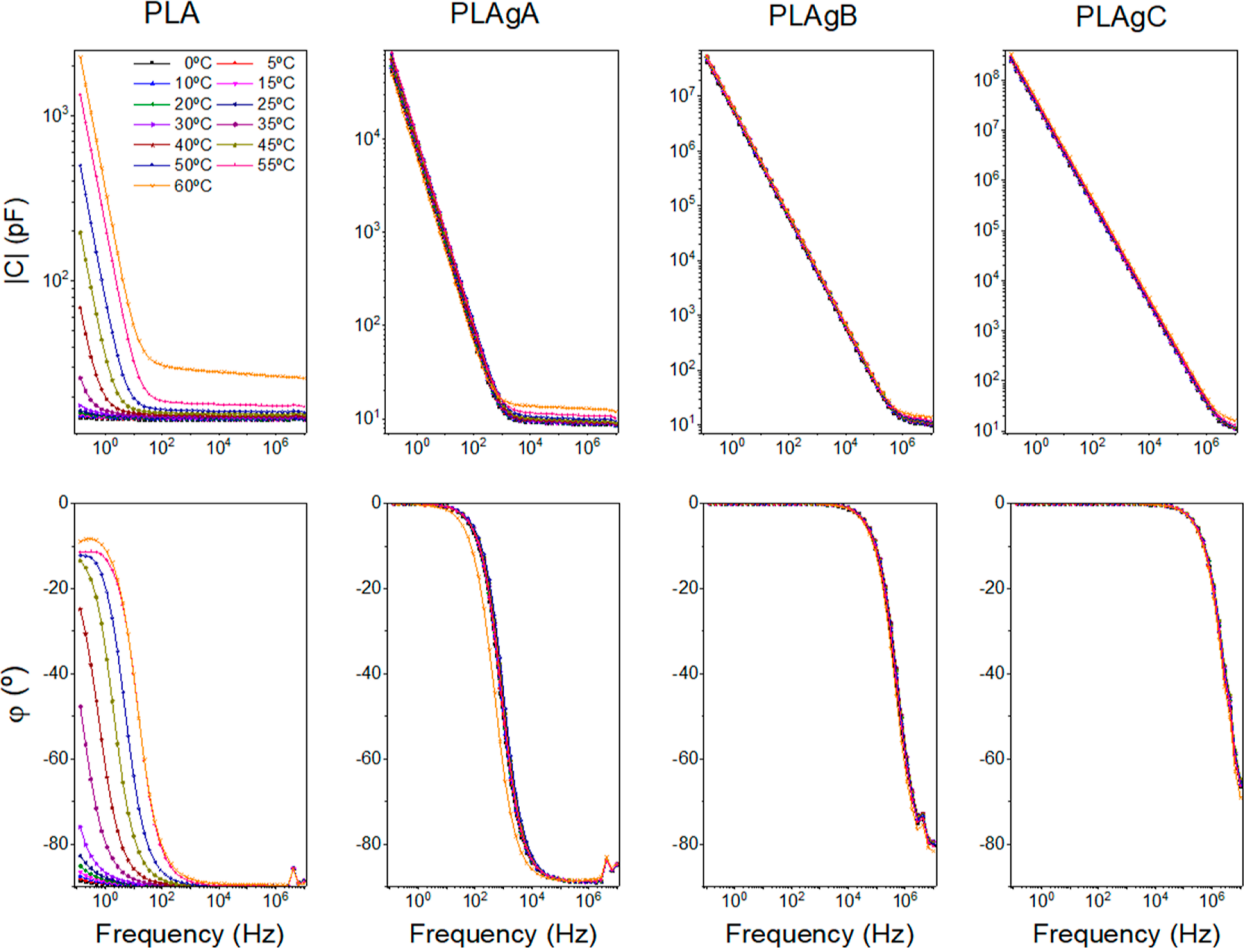
Geometrical capacitance as a function of the content of CNTs in PLA-based materials. Up: Modulus of capacity vs frequency at different temperatures. Down: phase angle vs frequency at different temperatures.

The values obtained for the modulus of capacity (|*C*|), are plotted in Figure 7. It can be observed that for the PLA-CNTs groups the modulus of capacity is directly proportional to the frequency, with a slope of about 1 (correlation coefficient ca. 0.999) for all temperatures. This can be of great interest in the case of materials used as electrolytes for capacitors and supercapacitors.^93^

From the results shown in Figure 7, we have calculated the capacitance, *C* = |*C*|(φ → −90°) for the PLA and PLA-CNTs samples. Notice that when the mass fraction of CNTs is low and intermediate (PLAgA and PLAgB), the tendency in the behavior of the samples is to change from a pure resistor to a dual behavior (resistor to capacitor) depending on the frequency interval to which the electric field is subjected on the material. For all samples, a capacitor behavior was observed at the high frequencies’ region observing that the critical frequency depends on the content in CNTs. The values obtained for each temperature are plotted in Figure S7. From this figure, we can conclude that the capacitance is practically constant with temperature in the range between 0 and 55 °C, following the trend *C* (PLAgA) = 9 pF < *C* (PLAgB) ≅ *C* (PLAgC) = 10 pF < *C* (PLA) = 14.5 pF.

**Figure 8:**
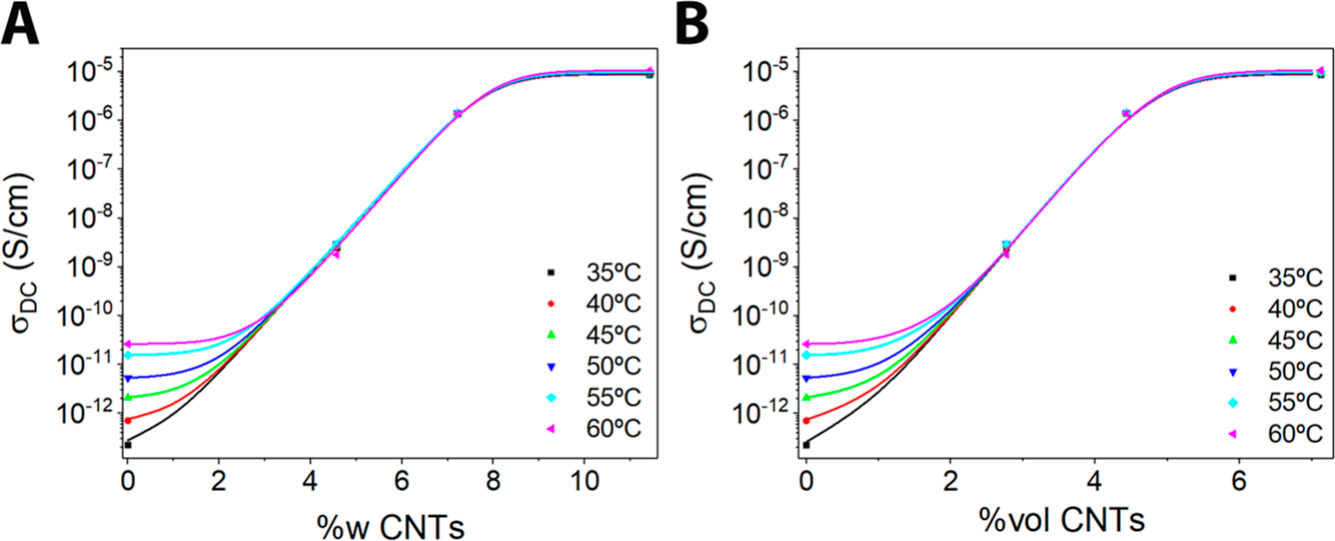
(A) Experimental electrical conductivity vs the mass fraction of CNTs present in the PLA membranes, measured at different temperatures. (B) Experimental electrical conductivity vs the volume fraction of CNTs present in the PLA membranes, measured at different temperatures. The symbols represent the experimental values, and the lines represent the fitting curves, applying eq 7.

Earlier, we have observed that increasing the content on CNTs of the PLA-CNTs samples increases the electrical conductivity. Figure 8A shows the relationship between the conductivity and the mass fraction of CNTs added to the PLA membranes. These curves show the experimental values of the conductivity versus mass fraction, together with the fitted curves calculated using a Boltzmann sigmoid, according to eq 7, where σ_0,_ and σ_∞_ are the values of electrical conductivity before and after the transition, *c* is an inflection point which represents the percolation threshold, and α is a coefficient that describes the behavior of the slope of the process during the transition and identifies the continuity or discontinuity of the process. The error of the fit to the experimental conductivity values is *R*^2^ = 0.985. From this figure, we can observe that the inflection point corresponding to the percolation threshold is achieved for a mass fraction of CNTs around 6%.
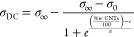
7

Percolation theory relates the conductivity of the composite to the volume fraction of the filler with the well-known eq 8,^94^ where *f* is the volume fraction, *f*_c_ is the amount of percolation threshold, and *t* is an exponent that explains the mechanism of network formation. The variation of conductivity as a function of the filler volumetric fraction is similar to the variation with respect to the filler mass fraction (Figure 8B). The obtained value of t is 1.97 ± 0.02 in a temperature range from 0 to 55 C and 2.09 for 60 C. This is indicative that the mechanism of network formation changes to 60 C, such as we can see from Figures 6 and7 for the PLA and PLAgC, respectively, as a consequence of glass transition which starts at about 53 °C (see Figure 3B). The value of *t* is consistent with other CNT-doped composites.^95^
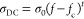
8

Regarding the dependence of the conductivity vs concentration of the CNTs grafted in the PLA fibers, measured at different temperatures, our study might suggest that about a 6% mass fraction of CNTs or 3% vol of CNTs could be the characteristic values of the percolation threshold. However, our study is limited due to the low number of experimental samples.

## 4 Conclusions

The results that we have obtained prove the successful production of a conductive material in the form of a nanofiber membrane, where CNTs play a fundamental role in providing enhanced mechanical and electroconductive properties, while PLA acts as host polymer and provides a biodegradable core, obtaining an environmentally friendly CPC. In addition, the nanofiber composition offers an excellent basis for directing electrical current and increasing the specific surface area, increasing the surface available to anchor the CNTs. The treatment with H_2_SO_4_ of PLA nanofibers substrates favors the union of functionalized CNTs with the carboxyl group, the treatment time and the drying of the substrates before the grafting with CNTs being critical, influencing the final properties of the materials. On the one hand, CNTs provide excellent mechanical properties, allowing the nanofibers to have greater rigidity, increasing the manipulability of the membranes and the preservation of their conformation when subjected to different stresses. On the other hand, grafting with CNTs allows us to eliminate the insulating barrier of the PLA, reducing its resistivity, and providing high electrical conductivity to the substrates. Thus, this is a facile method to improve the performance of PLA nanofiber-based materials by using CNTs that can be applied in the medical and industrial fields.

## Supporting Information Available

Transmittance ratios of treated and grafted samples regarding PLA; initial crystallinity (*X*_0_) obtained from DSC thermograms; ratios between transmittance values for most important wavenumbers; Nyquist diagram for the complex impedance spectra of PLA and PLA-CNTs samples at different temperatures; equivalent circuit proposed to fit the experimental values obtained for the imaginary and real part of the impedance of the samples studied; fit parameters obtained from the fit to the experimental data using the equivalent circuit; temperature dependence of *R*_p_, a, and *C*_p_ for PLA and PLA-CNTs samples; temperature dependence of the dc-conductivity for PLA and PLA-CNTs samples; and variation of the capacity versus temperature for the PLA and PLA-CNTs samples (PDF)
