# Supplementary material for: Polylactic Acid Nanofiber Membranes Grafted with Carbon Nanotubes with Enhanced Mechanical and Electrical Properties
Source: ACS Appl Polym Mater. 2023 Jul 12;5(8):6081–94. doi: 10.1021/acsapm.3c00776 (PMC10852358; doi:10.1021/acsapm.3c00776)
Supplement: Supplementary file 1 — ap3c00776_si_001.pdf [file ap3c00776_si_001.pdf]

Supporting Information

Polylactic Acid Nanofiber Membranes Grafted with Carbon Nanotubes with Enhanced Mechanical and Electrical Properties

Fernando Gisbert Roca [‡, a, *], *Cristina Martínez-Ramos* [‡, a, d], *Sergiy Ivashchenko* [‡, a], *Abel García-Bernabé* [b], *Vicente Compañ* [b] *and Manuel Monleón Pradas* [a,c]

[‡] These authors contributed equally.

[a] Center for Biomaterials and Tissue Engineering. Universitat Politècnica de València. Camino de Vera s/n, Valencia 46022, Spain.

[b] Departamento de Termodinámica Aplicada. Universitat Politècnica de València. Camino de Vera s/n, Valencia 46022, Spain.

[c] CIBER-BBN. Biomedical Research Networking Center in Bioengineering Biomaterials and Nanomedicine. Madrid 28029, Spain.

[d] Unitat Predepartamental de Medicina, Universitat Jaume I, 12071 Castellón de la Plana, Spain.

[*] Correspondence: ferrogi1@upv.es; Tel.: +34-963-877000

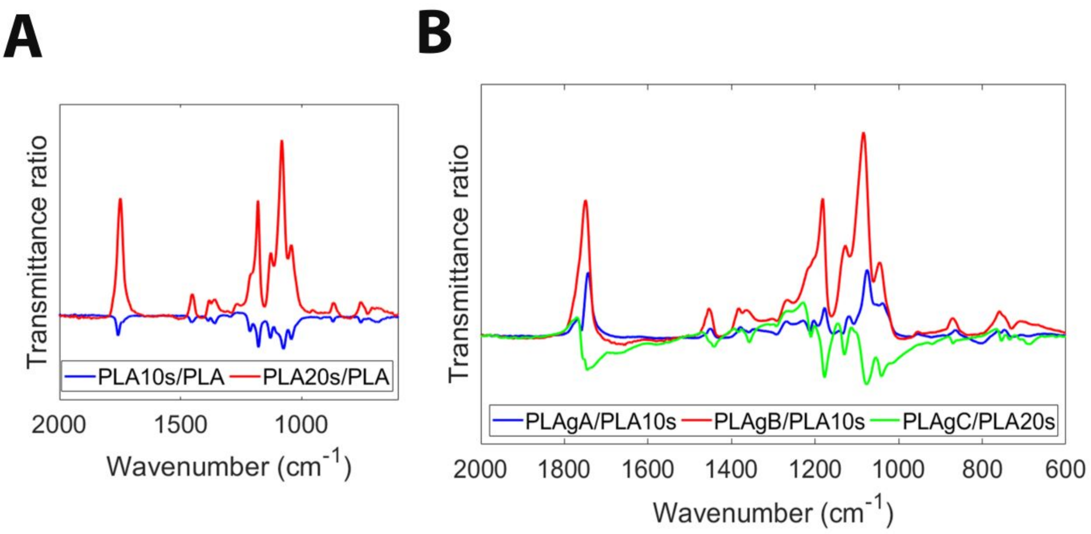

Figure S1. A: Transmittance ratios of treated samples (PLA10s and PLA20s) regarding PLA. **B**: Transmittance ratios of grafted samples (PLAgA, PLAgB and PLAgC) regarding the correspondent treated ones.

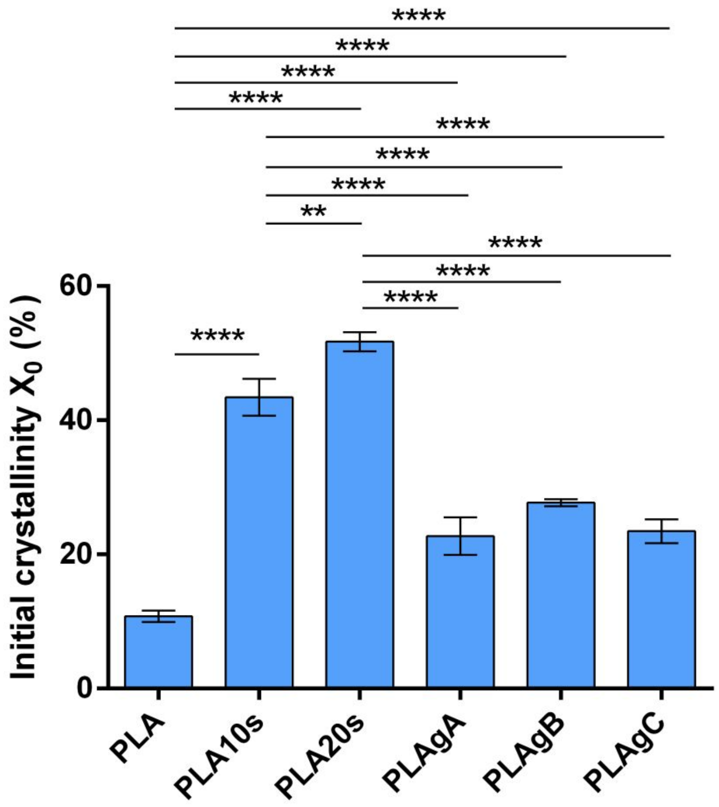

Figure S2. Initial crystallinity (X_0) of PLA, PLA10s, PLA20s, PLAgA, PLAgB and PLAgB obtained from DSC thermograms.

Table S1. Ratios between transmittance values for most important wavenumbers.

Wave-number, cm^{-1}	PLA peaks	PLA_10s/ neat PLA	PLA_20s/ neat PLA	PLA_gA / PLA_10s	PLA_gB / PLA_10s	PLA_gC / PLA_20s
1048	Symmetric C–O stretch (ether)	0.97	1.08	1.09	1.21	0.89
1092	Asymmetric C–O stretch (ether)	0.97	1.17	1.09	1.53	0.93
1132	Symmetric C–O stretch (ester)	0.97	1.07	1.03	1.25	0.95
1184	Asymmetric C–O stretch (ester)	0.96	1.14	1.08	1.38	0.89
1364	CH_3 symmetric deformation	0.99	1.02	1.02	1.07	1.01
1384	CH_3 symmetric deformation	0.99	1.02	1.03	1.08	1.02
1456	CH_3 asymmetric deformation	0.99	1.03	1.02	1.08	0.98
1759	C=O stretch	0.98	1.12	1.06	1.34	1.02

The values of the conductivity obtained from the Nyquist plot, also permit us to calculate the conductivity from the intercept in OX-axes determine the real part of the impedance which using the expression $R = {}^{L}/_{(A\sigma')}$ we obtain the real part of the conductivity (1). The Nyquist plots of the samples PLA, PLAgA, PLAgB and PLAgC at different temperatures are given in **Figure S3**. A clear semicircle followed by an accumulation of points when the imaginary part of the impedance tends to a zero value is observed for the samples PLAgA, PLAgB and PLAgC. However, depending on temperature the sample PLA shows depressed semicircles, or the semicircle curve disappeared leaving only the trend of the points towards low frequencies that can be explained by the increasing of the resistance of this polymeric film indicating that conductivity will be mainly due to the hopping of the impurities or ions present into the sample. The distribution of the relaxation times causes the depression of the semicircles. The typical equivalent circuit of a semicircle Nyquist plot consists of a capacitor in parallel with a resistor. When the semicircle is more depressed, the capacitor is replaced

by a constant phase element (CPE). **Figure S3** shows the representation of the imaginary part of the impedance versus the real part of the impedance (Z" (Ω) vs. Z' (Ω)), corresponding to the Nyquist plot for all the samples in the complete range of temperatures studied. The equivalent circuit to fit the experimental values following the equation SI1 is given in **Figure S4**.

$$Z^*(\omega) = \frac{R_p}{1 + R_p C_p (j\omega)^a} \quad \text{(SI1)}$$

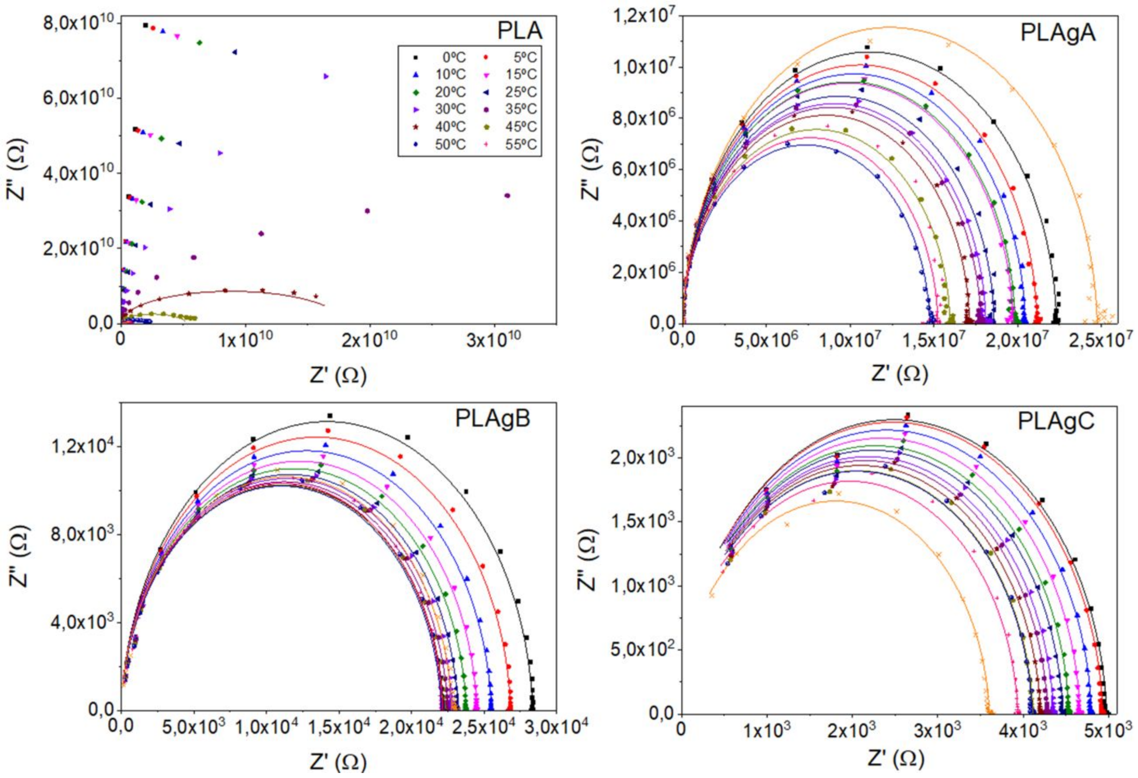

Figure S3. Nyquist diagram for the complex impedance spectra of PLA and PLA-CNTs samples at different temperatures.

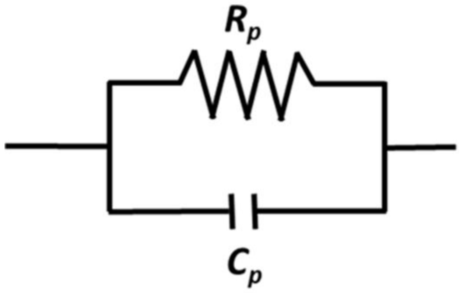

Figure S4. Equivalent circuit proposed to fit the experimental values obtained for the imaginary and real part of the impedance of the samples studied.

Table S2. Fit parameters obtained from the fit to the experimental data using the equivalent circuit represented in Figure S2.

T (°C)	Rp (Ω)	Cp (pF)	a	Rp (Ω)	Cp (pF)	a	Rp (Ω)	Cp (pF)	a	Rp (Ω)	Cp (pF)	a
**muestra**	**PLA**	**PLA**	**PLA**	**PLAgA**	**PLAgA**	**PLAgA**	**PLAgB**	**PLAgB**	**PLAgB**	**PLAgC**	**PLAgC**	**PLAgC**
0				2,23E+07	11,0	0,984	2,83E+04	17,9	0,969	4,98E+03	20,5	0,965
5				2,12E+07	10,7	0,987	2,68E+04	18,1	0,969	4,94E+03	19,8	0,965
10				2,04E+07	10,8	0,987	2,55E+04	18,3	0,969	4,80E+03	19,7	0,965
15				1,97E+07	10,9	0,986	2,45E+04	18,5	0,968	4,67E+03	19,7	0,965
20				1,98E+07	11,1	0,985	2,38E+04	18,8	0,968	4,54E+03	19,5	0,965
25				1,86E+07	10,9	0,988	2,32E+04	18,9	0,968	4,47E+03	19,2	0,965
30				1,81E+07	11,4	0,984	2,28E+04	18,9	0,968	4,36E+03	19,6	0,965
35				1,78E+07	11,5	0,984	2,25E+04	19,1	0,968	4,29E+03	19,8	0,965
40	1,78E+10	19,7	0,997	1,71E+07	11,8	0,984	2,22E+04	19,2	0,968	4,21E+03	20,1	0,965
45	5,20E+09	17,4	0,998	1,60E+07	12,1	0,984	2,21E+04	19,3	0,968	4,12E+03	20,5	0,965
50	1,98E+09	19,4	0,997	1,48E+07	13,0	0,981	2,21E+04	19,4	0,968	4,11E+03	20,6	0,965
55	6,35E+08	21,5	0,996	1,52E+07	13,8	0,986	2,21E+04	19,7	0,975	3,94E+03	22,4	0,965
60	4,11E+08	41,2	0,956	2,48E+07	18,3	0,973	2,28E+04	22,6	0,974	3,60E+03	28,1	0,965

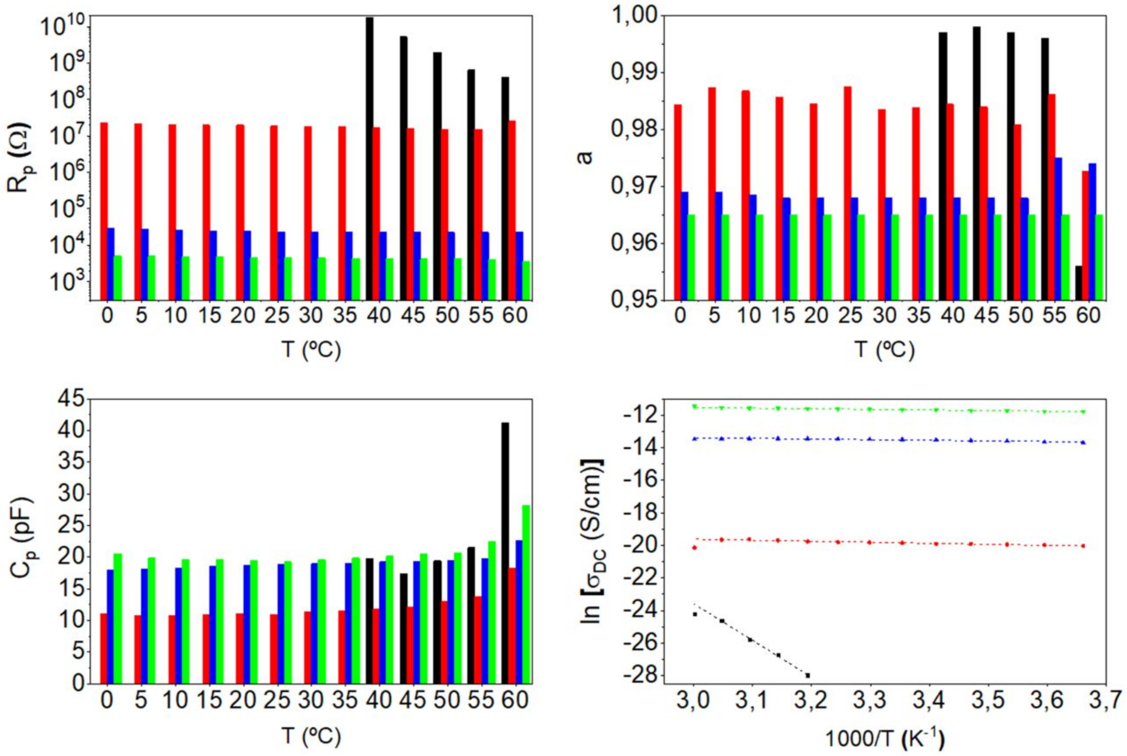

Figure S5. Temperature dependence of R_p, a and C_p for all the samples studied PLA (■), PLAgA (●), PLAgB (▲) and PLAgC (▼), respectively.

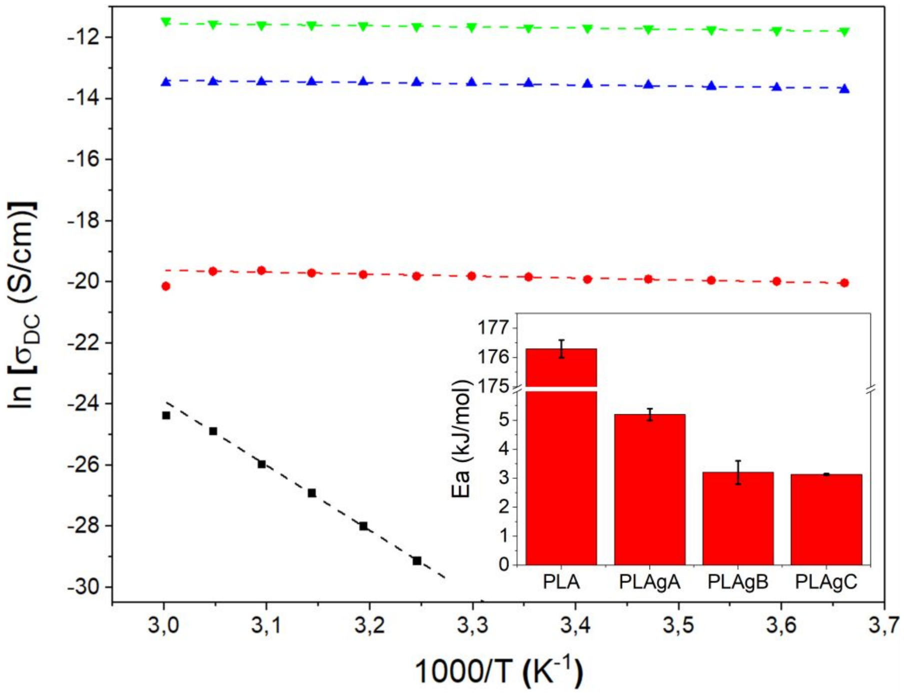

Figure S6. Temperature dependence of the dc-conductivity for: PLA (■), PLAgA (●), PLAgB (▲) and PLAgC (▼), respectively. The Inset correspond to the activation energy (Ea) values obtained from the fit of the Arrhenius plot of the conductivity obtained from the Bode plots.

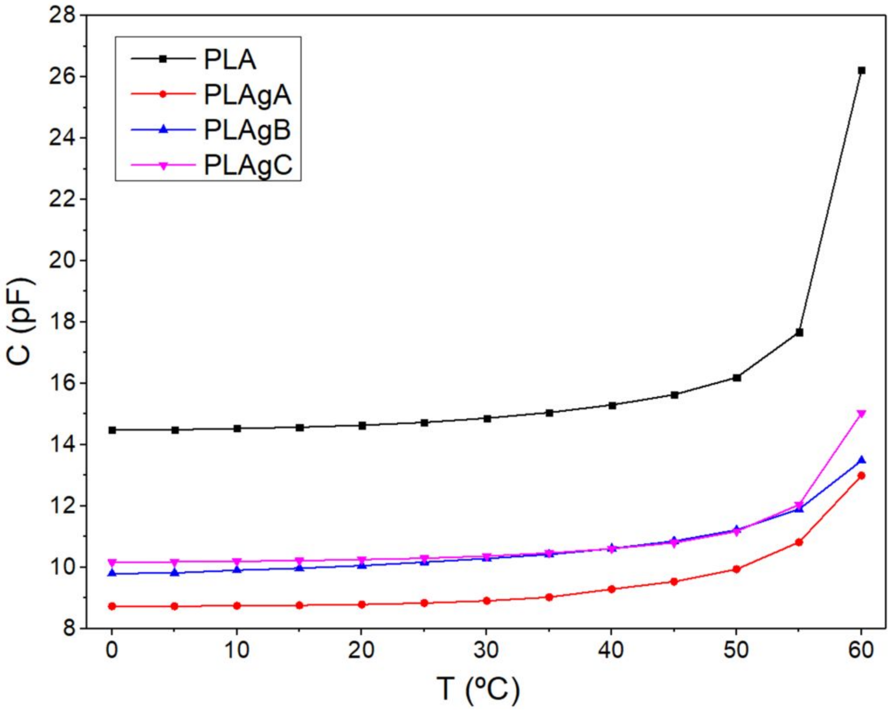

Figure S7. Variation of the capacity versus temperature for the PLA and PLA-CNTs samples.

References

1. V. Compañ, J. Escorihuela, J. Olvera, A. Garcia-Bernabe, A. Andrio. Influence of the anion on diffusivity and mobility of ionic liquids composite polybenzimidazol membranes. Electrochimica Acta 2020, 354, 136666.
